# The Part Which Vital Action Plays in the History of Dental Caries

**Published:** 1874-10

**Authors:** H. S. Chase

**Affiliations:** St. Louis.


### ARTICLE III.

### *The Part which Vital Action Plays in the History of Dental Caries.*

BY DR. H. S. CHASE, ST. LOUIS.

Read before the Illinois State Dental Society.

The various theories in regard to the kind or kinds of action which constitute dental caries, have probably more or less of truth in them all. That the process which produces that disintegration of the dental tissues which is usually called by the name of dental caries, is wholly chemical, or wholly vital, I do not believe. Neither do I think that there is sufficient evidence that fungi assist materially in its development and progress. The great number of observations which have been made in regard to the action which acids have in the disintegration of the dental tissues, have well established the fact that the destruction of tooth substance is mainly produced by chemical decomposition.

I think that the greater number of the members of the dental profession believe that chemical decomposition is the *sole* process in the history of dental decay or dental caries.

*Formerly* vital action had greater consideration in the production of this disease than it has at the present time; and it is my object in this paper to present a few thoughts in favor of the vital movements which I believe undoubtedly play an important part in the progress of dental disintegration, and to give to those vital movements a place in the history of this disease which I believe they deserve.

Many persons assume that all vital movements cease in the dentine and cementum when those tissues are fully formed. A still larger number declare that there is no vital action whatever in the enamel substance when *that* is fully formed. I believe that observation will establish facts that will prove these assumptions to be false.

Admitted facts prove that vital processes are the sole cause of the production and completion of all the hard tissues of the teeth. And the history of the shedding of the temporary or milk teeth prove that it is vital action alone which removes the roots and whole body of the dentinal crown. It is easily seen, then, that it is a living movement that builds up the tooth structure to its highest perfection, and also as patent that it is the same process, in a different direction, that takes down, particle by particle, or cell by cell, that same edifice which was so beautifully erected.

Microscopical observations have proven that a barrier of defense is often interposed between a cavity of decay and the dental pulp by an increased calcification of the dentine at that particular location, resulting in an increased hardness and density of the dentine. Here is a vital process again; and this action takes place in the teeth of the middle aged, as well as in those of the young.

Again, in cases of mechanical abrasion, where the teeth are worn down below the thickness of the enamel, we often find the surface of the dentine almost as hard and polished as the enamel itself; and this density of the dentine does not extend to a great depth, but on the contrary, is generally limited to a thin shell of that portion of dentine which is liable to the destructive abrasion, looking very much as though the tooth was endeavoring to protect itself by packing its tubuli with calciferous matter, and thereby forming a wall of defense.

Some of the most careful observers declare that they have known, in their own experience, an absorption of dentine, under a perfect plug, to take place to the extent of exposing the dental pulp; the exposure taking place absolutely without dental decay, and to such an extent that there could be no mistake about it.

A large number of intelligent men are quite sure, from a great number of observations, extending over many years of experience, that the permanent teeth of the adult may become less dense in the structure of the dentine than the same teeth were at a former period; in fact, an interstitial decalcification of that tissue having taken place.

These facts prove, then, that the two opposite processes of calcification and decalcification may, and do take place, in the perfected hard tissues of the teeth, and furthermore, that the basal structure itself, or the animal portion of dentine, may be removed without chemical action.

Beside the foregoing facts, I wish to describe a condition of the enamel and dentine which I have very many times observed, namely: a black or brownish spot is seen on the proximate surface of a molar or bicuspid tooth; it is smooth, hard and glossy; in fact, seems to have as dense a *surface* as glass, or as the uncolored and perfect enamel on the same tooth. A fine excavator, in passing over it, detects no abrasion or disintegration. On cutting into this discolored portion, the tissue appears as solid, for a little distance, as ordinary enamel. The *dentinal* surface of this enamel is somewhat lighter colored than the exterior. As soon as the *dentine* is reached, we observe a softer condition, but the dentine is not discolored, still it seems somewhat decalcified. On proceeding a little further, we find this tissue still softer, caused, apparently, by a *greater* decalcification, and then we come again to normally hard dentine. In all this there is evidently no loss of *basal* structure, either of enamel or dentine, but there is a loss of *substance*, and this is in the *lime* salts of the dentine. It is apparent that there has been *external* loss of tooth substance; there has been no chemical process taking place; but there has been a vital movement in the interior of the tooth; in fact, among the tubuli themselves, resulting in the process called absorption, the calcareous elements having been carried away through the interior vessels of the tooth, and not by a chemical wasting away from the exterior.

A progressive history of this diseased condition would result in showing, that after a longer period the exterior surface would show signs of disintegration; the surface would become roughened; little pits would be found in it, and on cutting it with an excavator the enamel would be found softened and granular; it would easily break down under the instrument, and show discoloration as deep on its dentinal surface as on its exterior. Beneath the enamel the dentine would show a brownish discoloration, and an entire decalcification for a greater or lesser distance, until the normally hard and healthy dentine is reached.

Now, in this subsequent history of this disease, we probably have more or less chemical action to account for, and an *exterior* loss of substance produced by it. And this exterior chemical action of acids on the enamel will undoubtedly progress toward the interior or dentinal portion of the tooth, as rapidily as a fresh surface is presented by the washing or wasting away of the decomposed tissues.

Pertinent to the subject would be the question: What has caused the condition of things corresponding to the *earlier* history of the tooth described? I answer, *necrosis* of the enamel was produced by pressure of a contiguous tooth, causing the circulation of the blood plasma to cease in the basal substance of the enamel, and discoloration taking place as a consequence of necrosis, as usual. The irritation or inflammation consequent on or simultaneous with this necrotic process caused the vital movement in the dentine which carried away its lime salts.

It must be remembered that irritation of a tissue may produce under, apparently, the same circumstances, either of two opposite vital movements, namely: a loss of substance, or an increase of substance. This is known to be the case in the bones of the body and in its softer tissues. In the teeth themselves exostosis is caused by the irritation of a dead and decomposing pulp, at other times absorption of the cementum and dentine of the roots takes place from the same cause.

I have already instanced the absorption of dentine under a plug which had irritated the dental pulp by the thermal changes produced by the conducting powers of the metal. At other times the same irritation produced by the same cause calls into activity those vital movements which protect the pulp by a new deposit of dental tissue between the pulp and the plug.

And here we will call to mind, again, that result of vital activity which interposes a denser wall of dentine around a carious cavity in which chemical processes were in the active destruction of tooth substance.

With these facts before us, is it not probable that in many cases of chemical disintegration of the teeth, the irritation produced in the dental tissues causes a vital movement, resulting in rapid decalcification of the dentine, and perhaps also, absorption of the basal or animal substance of that tissue, instead of the opposite process of extra deposition of lime salts in the dentine as a barrier to the decaying process?

It seems to me, from the foregoing observations, that taking a broad view of the process of dental caries, we must give to vital action an important place in its history.

In conclusion, I wish to say that I have read Dr. Chandler's translation of Leber and Rottenstein's Researches on Dental Caries. These authors give great importance to influence produced by the Leptothrix Buccalis, which they say is almost invariably found in all carious dentine and enamel. This fungus is also found upon the tongues and upon the gums of persons who are not particular in cleansing the mouth daily. My conclusions, taking their *observations* as correct, which I believe they are, lead me to consider the Leptothrix as an agent which cannot initiate dental caries, but as a concomitant of the disease, may accelerate its progress. And I would here take occasion to heartily recommend the work of these authors on this subject to my professional brethren.

